# High-sensitivity piezoelectric perovskites for magnetoelectric composites

**DOI:** 10.1088/1468-6996/16/1/016001

**Published:** 2015-02-18

**Authors:** Harvey Amorín, Miguel Algueró, Rubén Del Campo, Eladio Vila, Pablo Ramos, Mickael Dollé, Yonny Romaguera-Barcelay, Javier Pérez De La Cruz, Alicia Castro

**Affiliations:** 1 Instituto de Ciencia de Materiales de Madrid, CSIC, Cantoblanco, 28049 Madrid, Spain; 2 Universidad de Alcalá, 28871 Alcalá de Henares, Spain; 3 Département de Chimie, Université de Montréal C.P. 6128, succursale Centre-Ville Montréal, QC, H3C 3J7, Canada; 4 IFIMUP and IN-Institute of Nanoscience and Nanotechnology, Faculdade de Ciências da Universidade do Porto, Rua do Campo Alegre, 687, 4169-007 Porto, Portugal

**Keywords:** perovskite oxides, piezoelectrics, magnetoelectrics, ceramic composites

## Abstract

A highly topical set of perovskite oxides are high-sensitivity piezoelectric ones, among which Pb(Zr,Ti)O_3_ at the morphotropic phase boundary (MPB) between ferroelectric rhombohedral and tetragonal polymorphic phases is reckoned a case study. Piezoelectric ceramics are used in a wide range of mature, electromechanical transduction technologies like piezoelectric sensors, actuators and ultrasound generation, to name only a few examples, and more recently for demonstrating novel applications like magnetoelectric composites. In this case, piezoelectric perovskites are combined with magnetostrictive materials to provide magnetoelectricity as a product property of the piezoelectricity and piezomagnetism of the component phases. Interfaces play a key issue, for they control the mechanical coupling between the piezoresponsive phases. We present here main results of our investigation on the suitability of the high sensitivity MPB piezoelectric perovskite BiScO_3_–PbTiO_3_ in combination with ferrimagnetic spinel oxides for magnetoelectric composites. Emphasis has been put on the processing at low temperature to control reactions and interdiffusion between the two oxides. The role of the grain size effects is extensively addressed.

## 1. Introduction

Piezoelectric materials develop a linear electrical polarization in response to a mechanical stress: the direct piezoelectric effect, and linearly deform under the application of an electric field: the converse piezoelectric effect [1]. They are thus also electromechanical transducers, and show enhanced conversion efficiency at mechanical resonance frequencies. Piezoelectric ceramics are a mature and ubiquitous technology, which is the basis of a wide range of applications, such as acceleration sensors, flow meters, actuators (fuel injectors, positioning systems, etc), smart systems (active control of vibrations and structures, adaptive optics, etc), ultrasound transducers (either for non destructive testing or for medical imaging), underwater acoustics, and many others [2–4]. Currently, they are also being considered for novel applications like energy harvesting and magnetoelectric transduction [5, 6].

High-sensitivity piezoelectrics are mostly perovskite ABO_3_ oxides. The current state of the art material is Pb(Zr,Ti)O_3_ (PZT) at the morphotropic phase boundary (MPB) between the ferroelectric rhombohedral *R*3*m* and tetragonal *P*4*mm* polymorphs [1–4]. It is acknowledged that ferroelectrics at phase instability regions and the presence of monoclinic phases between the rhombohedral and tetragonal ones are key to high piezoelectric response [7, 8]. Domain dynamics also play a major role, and indeed, large piezoelectric coefficients can be achieved by enhancing the ferroelectric/ferroelastic domain walls mobility [9]. A main line of research at the cutting edge of the field is the design and synthesis of novel perovskite systems with ferroelectric MPBs and high piezoelectric response that can replace PZT, either lead-free, necessary for environmentally friendly piezoelectric elements [10], or high Curie temperature *T*_C_ to enable electromechanical transduction in harsh environments [11].

Bi-containing perovskites are playing a major role in both topics, though advances are hindered by thermodynamic instability associated with a low tolerance factor, which causes a number of very promising systems to be obtained only by high-pressure synthesis [12]. Mechanochemical activation is being considered an alternative route to obtain low-tolerance factor perovskites that cannot be obtained by conventional synthesis routes, and indeed we have succeeded in mechanosynthesizing a number of examples [13–15]. The solid solution of (1–*x*)BiScO_3_–(*x*)PbTiO_3_ is receiving a lot of attention as an alternative system to PZT for the next generation of high-temperature and high-sensitivity piezoelectric devices. This system exhibits large piezoelectric coefficient *d*_33_ of ∼450 pC N^−1^ and high *T*_C_ of ∼450 °C [16] (this is 100 °C above that of PZT), and better properties on grain size down-scaling [17–19]. As expected, best properties are found around a MPB (for *x* ∼ 0.64) analogous to that of PZT [20].

Perovskite MPB piezoelectrics are being considered in combination with magnetostrictive materials for the development of magnetoelectric composites, in which magnetoelectricity is obtained as a product tensor property of the piezoelectricity and effective piezomagnetism of the two ferroic phases [21–23]. Direct and converse effects also result: the development of an electric polarization **P** proportional to an applied magnetic field **H** and of magnetization **M** in response to an applied electric field **E**, respectively. This opens the possibility of enabling a range of novel disruptive technologies, such as: high-sensitivity magnetic field sensors with room-temperature (RT) operation, microgenerators for remote powering of wireless bio-implanted devices from ambient or directed magnetic fields, or dual electric-field magnetic-field tunable microwave devices such as filters, resonators and phase shifters [24–26].

Perovskite PZT is the usual choice for the piezoelectric phase, while magnetostrictive phases could be either metallic alloys or magnetic oxides [21–23]. These latter materials are not actual piezomagnetics, but effective responses can be obtained under bias magnetic field. A range of two-phase composites with 0–3 and 2–2 connectivities have been demonstrated, among which laminate composites fabricated by the epoxy-bonding method with modified PZT and giant magnetostrictive metal alloys like tefenol-D and metglas stand out [27]. However, this method presents a challenge for mass fabrication, and cofiring techniques to obtain direct interfaces would be preferred. In this case, research focuses in all-oxide ceramic composites obtained by co-sintering at high temperatures, among which ferrimagnetic spinels and ferroelectric perovskite materials play a major role, with magnetoelectric coefficients of several orders of magnitudes above those shown by single-phase multiferroics [28–30].

Nevertheless, the magnetoelectric effects so far observed in high-temperature cofired bulk ceramic composites are still much lower than those theoretically predicted, due mainly to the poor mechanical coupling between the phases and defects concentrated at the interfaces [22]. The novel functionality in these two-phase composite oxides is a strain-mediated effect, so a major issue is to optimize strain continuity across the piezoelectric–magnetic interfaces, and a main line of research is controlling diffusion phenomena across and chemical reactions at the interfaces during high-temperature preparation.

In this context, we are investigating novel methods for the preparation of laminate ceramic composites with high-quality interfaces, by using nanopowders of the ferroic oxides obtained by mechanosynthesis and wet-chemistry routes, and cofiring them by spark plasma sintering. We present here results on the suitability of using the high-sensitivity piezoelectric perovskite BiScO_3_–PbTiO_3_ in combination with the ferrimagnetic spinel NiFe_2_O_4_ for the preparation of multilayer magnetoelectric composites made of alternating piezoelectric and magnetostrictive layers obtained by tape-casting. Emphasis has been put on controlling chemical reactions and interdiffusion across the interfaces to obtain high magnetoelectric response. Microstructural and interface features are described, and the main issues relevant to functionality are discussed.

## 2. Experimental procedures

**Figure 1.**
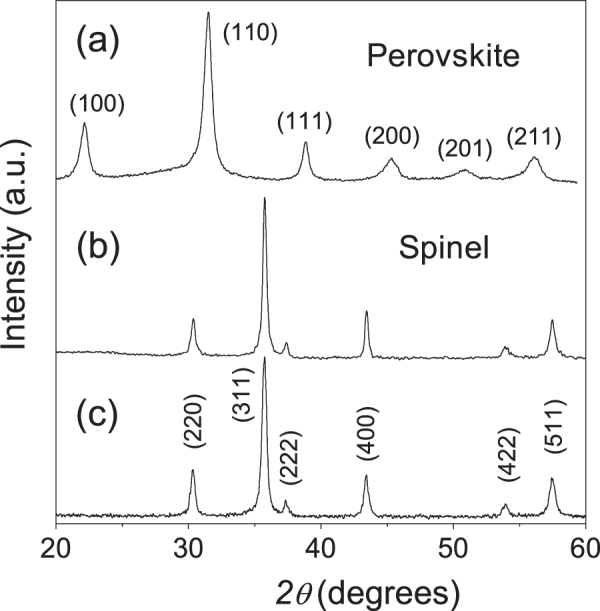
XRD patterns corresponding to (a) the 0.36BiScO_3_–0.64PbTiO_3_ perovskite phase; and those for the NiFe_2_O_4_ spinel phases obtained by (b) mechanochemical activation and (c) wet-chemistry, after annealing at 600 °C.

Perovskite phase nanocrystalline powders of 0.36BiScO_3_–0.64PbTiO_3_ (BSPT) were obtained by mechanochemical treatment of stoichiometric mixtures of analytical grade Bi_2_O_3_, Sc_2_O_3_, PbO and TiO_2_, with a Pulverisette 6 (Fritsch) planetary mill. The mechanosynthesis of the perovskite as single phase was successfully achieved after only 20 h of milling, as shown in figure 1(a). An average particle size of 23 nm with standard deviation (SD) of 11 nm resulted, as measured by transmission electron microscopy (TEM) [31]. For the mechanochemical synthesis of the NiFe_2_O_4_, analytical grade NiO and Fe_2_O_3_ were used as starting reagents. The spinel phase could be completely isolated after a thermal treatment at 600 °C/2 h, as shown in figure 1(b). An average particle size of 30 nm with SD of 13 nm resulted. Details of the procedures and of the mechanisms taking place during the mechanosynthesis of these two systems, the perovskite and the spinel, can be found elsewhere [13, 31].

A wet-chemistry route was also employed for the synthesis of reactive precursors of the ferrite NiFe_2_O_4_, as described elsewhere [32]. Reagent grade 0.06 mole of Fe(NO_3_)_3_·9H_2_O and 0.03 mole of Ni(NO_3_)_2_·6H_2_O were dissolved in 300 ml of distilled water with 3 ml of HNO_3_ (65% concentration). Precursor materials were obtained by precipitation of Fe^3+^ and Ni^2+^ cations with dropwise addition (2 ml min^−1^) of 1 M solution of *n*-butylamine at RT, under vigorous stirring, up to pH = 10. Then, these suspensions were filtered, washed with distilled water, and dried at 80 °C. The solid precursors were annealed at 600 °C for removing water, nitrates, and organic ions, which allows the synthesis of the spinel as single phase, as shown in figure 1(c). In this case, an average particle size of 35 nm with SD of 16 nm resulted.

Tape casting was used to obtain laminate composites of alternating magnetostrictive and piezoelectric thick films. Ethanol-based slurries with 20 vol% of powders were developed to avoid the use of highly toxic toluene-based solvents [33]. The binder system was optimized to ensure similar organic volume fractions in the perovskite and spinel phases, to prevent delamination due to uneven shrinkage during organics burn out process. After drying, tapes with thickness of 35 to 50 *μ*m resulted, from which multilayer composites were prepared by lamination under uniaxial pressure of 10 MPa. Organics were burned out at 500 °C/2 h, with slow heating/cooling rates of 0.5 ºC min^−1^ to avoid delamination. Ceramic composites were prepared by spark plasma sintering (SPS, Dr Sinter 2080 Sumitomo apparatus) under uniaxial pressure of 100 MPa and sintering temperature between 700 and 1000 °C with heating rate of 375 °C min^−1^. The soaking time at the final temperatures and pressure was 10 min. Density was measured by Archimedes’s method in distilled water after polishing.

X-ray diffraction (XRD) was carried out with a Bruker D8 Advance diffractometer (CuK*α* radiation) in the range from 5 to 70° (2*θ*), in steps of 0.05° and counting time of 1.5 s/step. Ceramic microstructures were characterized by field emission scanning electron microscopy, FE-SEM (FEI Nova NanoSEM 230 microscope) on cross-sections perpendicular to the casting plane. Samples were prepared by polishing with Al_2_O_3_ suspensions down to 0.1 *μ*m. The Feret diameters of more than 300 grains were measured from SEM images to obtain reliable size distributions. The average size and SD were obtained with statistical analysis by using probability plots. Linear fits with correlation factor above 0.99 were obtained.

Electrical characterization was carried out on ceramic discs, on which Ag electrodes were painted and annealed at 700 °C/1 h. The temperature dependence of the dielectric permittivity and losses were dynamically measured under heating at 1.5 °C min^−1^, with an HP4284A precision LCR Meter in the frequency range 100 Hz–1 MHz. Polarization–electric field (P–E) ferroelectric hysteresis loops were obtained by current integration. High voltage sine waves (0.1 Hz) were applied by the combination of a synthesizer/function generator (HP3325B) and a high-voltage amplifier (Trek Model 10/40A), while charge was measured with a homebuilt charge to voltage converter. Loops are presented after subtracting linear polarization and conduction contributions from the current response, assuming a resistance and a capacitance in parallel. The *d*_33_ piezoelectric coefficient was measured with a Berlincourt-type piezometer.

Magnetic measurements were carried out with a Quantum Design MPMS-5S SQUID magnetometer. Isothermal magnetization–magnetic field (M–H) curves were recorded at RT with magnetic fields of ±3 kOe. The characterization of the magnetoelectric response was carried out with a system (Serviciencia SL) consisting of a combination of two Helmholtz coils: a high power coil and a high frequency one, designed to independently provide a dc magnetic field up to 1 kOe (to magnetize the material), and an ac magnetic field up to 10 Oe at 10 kHz (the stimulus). Magnetoelectric output voltages were monitored with a lock-in amplifier. The thickness of the piezoelectric element was used to compute the magnetoelectric coefficient, which allows comparing the response of trilayers and multilayers.

## 3. Results and discussion

The magnetoelectric response of laminated composites is determined by three main issues [26]: (i) the inherent properties of the constituent phases (e.g., dielectric permittivity and losses, elastic stiffness, piezoelectric and effective piezomagnetic coefficients, etc); (ii) the quality of the interfaces and geometry of the composites (e.g., pores, defects and stress along the interfaces, volume to thickness ratio of the layers, etc); and (iii) the operation mode (i.e., orientation of the applied magnetic field with respect to the alternating layers). For the latter, it is well-known that measuring the magnetoelectric effect in a longitudinal–transverse (L–T) field mode, in which the applied magnetic field is oriented parallel to the alternating layers whereas the sample is poled and the output voltage measured perpendicular to them, results in a much larger signal [22, 27]. On the other hand, the control of the functional properties of the constituent phases near the interfaces is as important as the effectiveness of the elastic coupling and strain continuity through them.

The processing method is key to obtain tailored interfaces in composites, and this usually requires low-temperature cofiring techniques, which also limits grain growth [28–30]. The unique features of the SPS makes possible to obtain fully dense materials at relatively low temperatures with controlled grain growth [34]. Besides, small grains at the interfaces would be advantageous for diminishing the thermal expansion mismatch between the dissimilar ceramic phases, thus reducing stress effects that may generate cracks and delamination. In such cases, however, it would be necessary to consider the effects of the grain size reduction on the functional properties of the perovskite phase, which may be an issue for obtaining large magnetoelectric response in low-temperature cofiring composites.

**Figure 2.**
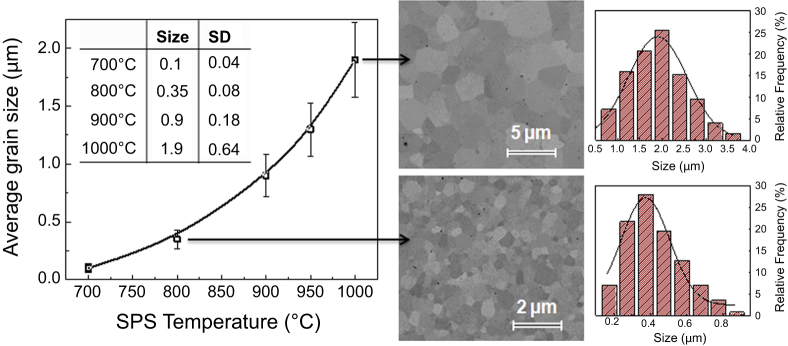
Evolution of the average grain size for the perovskite BSPT with the SPS temperature, average sizes and standard deviation (SD) are given. SEM images of polished surfaces of two representative samples along with their size distributions.

Grain size effects were studied for the functional properties of perovskite BSPT ceramics with grain sizes in the range obtained for the composites. Samples were processed by SPS under tailored conditions of nanopowders obtained by mechanosynthesis. Figure 2 shows the evolution of the average grain size with the SPS temperature, whose exponential increment suggests a single mechanism of grain growth. Error bars represent the SD obtained from the size distributions for each data point. SEM images of polished surfaces of two representative samples are also shown, along with the size distributions resulting, to illustrate the high-density and quality of the materials prepared. In these images, individual grains are distinguished by a gray scale map based on the electron backscattering diffraction pattern, in which gray contrasts correspond to changes in crystal orientations [35].

**Figure 3.**
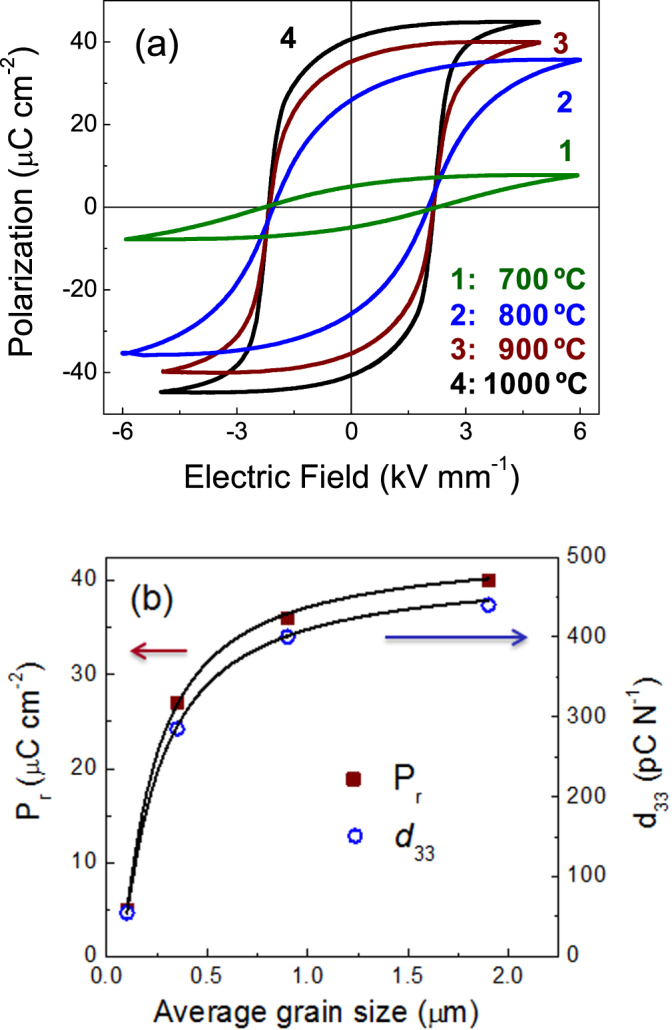
(a) Ferroelectric hysteresis loops for BiScO_3_–PbTiO_3_ ceramics obtained at different SPS temperatures, and (b) evolution of the remnant polarization (*P*_r_) and *d*_33_ piezoelectric coefficient with increasing grain size.

Figure 3(a) illustrates the grain size effect on the ferroelectric switching of these ceramics. Square hysteresis loops close to saturation of the polarization are obtained for ceramics with grain sizes about and above 1 *μ*m. Remnant polarization *P*_r_ slowly decreases from 40 to 36 *μ*C cm^−2^ when entering the submicron range, and then drops down to 27 *μ*C cm^−2^ as the grain size was reduced to 0.35 *μ*m, and only 5 *μ*C cm^−2^ was achieved for 0.1 *μ*m. Size effects were also observed in the piezoelectric charge coefficient *d*_33_ (figure 3(b)), which slowly decreases from 440 to 400 pC N^−1^ when entering the submicron range, and suddenly drops down to 285 and 55 pC N^−1^ for 0.35 and 0.10 *μ*m, respectively.

It has been recently shown that the grain size dependence of *P*_r_ (and also *d*_33_) actually reflects the increase of the effective coercitivity as the grain size decreases rather than a real decrease of the spontaneous polarization [36]. Higher electric fields would be required to approach saturation, and thus achieve an efficient poling (and a higher *d*_33_) of the material. However, infinitely high fields cannot be applied because electrical breakdown takes place (e.g., above 6 kV mm^−1^). The increase of the effective coercitivity resulting in the loss of functionality was associated with the presence of grain boundaries with reduced permittivity. The decrease of *d*_33_ with grain size thus reflects how one moves away from the saturation condition as the effective *E*_c_ increases with decreasing grain size, and an efficient poling of the material cannot be achieved. Therefore, small grain sizes in the perovskite component of laminated composites would be detrimental for the magnetoelectric response.

Perovskite/spinel/perovskite trilayers with 1 mm thickness each element were prepared, as they are especially suitable to study chemical reactions and interdiffusion at the interfaces, and for addressing compatibility issues during SPS of the component phases. The different shrinkage behaviour of these dissimilar phases may lead to cracking and delamination, and thus result in poor properties [29]. Results in terms of microstructure and properties are the same if the perovskite is the one sandwiched between spinel phases in the trilayers.

**Figure 4.**
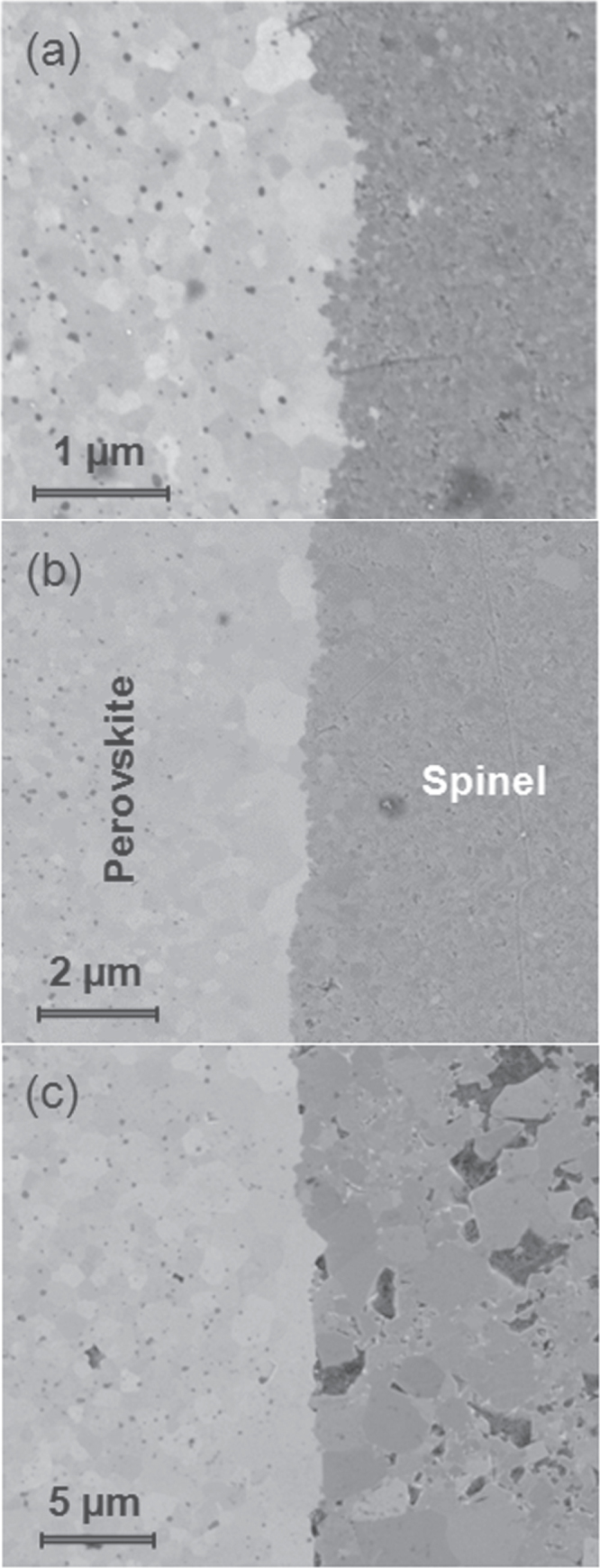
Representative SEM images of polished cross-sections at perovskite/spinel interfaces in trilayers prepared by SPS at (a) 800, (b) 900 and (c) 1000 °C, using the ferrite obtained by mechanochemical activation.

Figure 4 shows representative SEM images of polished cross-sections of trilayers prepared at 800, 900 and 1000 °C, showing the interface between the perovskite and spinel phases. Chemical reaction and interdiffusion phenomena at the interfaces were not detected, and nor was the occurrence of abnormal grain growth in the perovskite side, as previously reported for composites obtained by hot-pressing or conventional sintering [29, 31]. These abnormal grains were attributed to diffusion of ions from the spinel phase into the perovskite with diffusion lengths of 20 to 30 *μ*m, and thus should result in poorer piezoelectric properties.

A common feature of the samples at 800 and 900 °C is the high-quality of the interfaces in terms of the absence of cracks or delamination, where differences between them are basically in the grain sizes. Fairly dense microstructures with very few isolated pores were obtained. The composite prepared at 800 °C (figure 4(a)) shows negligible porosity and very small grain sizes approaching the nanoscale, for both phases. As expected, grain size increases with the SPS temperature. The composite prepared at 900 °C (figure 4(b)) shows submicron-sized grains in both phases, with a high-quality interface; whereas the sample prepared at 1000 °C (figure 4(c)) shows micron-sized grains, with the perovskite phase denser than the ferrite one. However, this sample showed a bimodal and degraded microstructure at the ferrite side, in which cracks and large voids can be observed. The densification of this trilayer decreased to less than 92% of the theoretical density (due basically to the porosity of the spinel phase), as compared to that about 99% for the other two samples (densification was calculated taking into account the characteristics of the trilayers). The degradation of the spinel when the sintering temperature reaches 1000 °C is the result of an abnormal grain growth.

**Figure 5.**
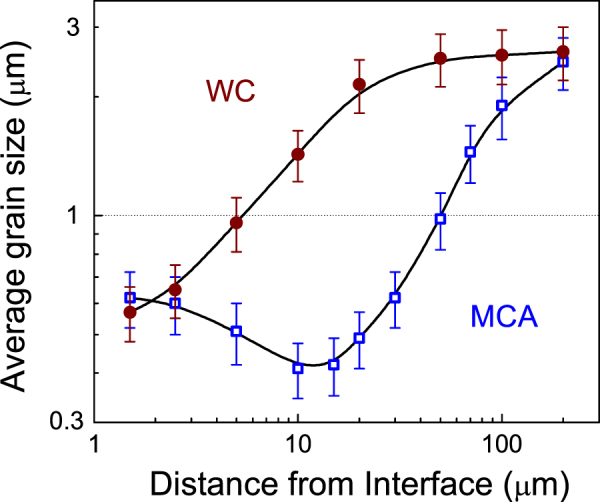
Evolution of the perovskite average grain size with the distance from the interface for trilayers prepared by SPS at 900 °C, using ferrites obtained by wet-chemistry (WC) and mechanochemical activation (MCA).

Nevertheless, none of the trilayers of figure 4 showed a homogeneous microstructure over the whole perovskite phase, and instead grain size gradients were obtained from the interface to the inner BSPT matrix, which spreads over a region that extends up to 200 *μ*m. Figure 5 shows the evolutions of the perovskite average grain size with distance from the interface for the trilayer prepared at 900 °C (curve with open dots). The plot is given in log–log scale for a better visualization of the data evolution, and the SD calculated from the size distributions are indicated as the error bars. The grain size shows a peculiar evolution with distance for this trilayer prepared with the spinel obtained by mechanochemical synthesis, for which a minimum average size of ∼0.4 *μ*m was achieved at about 10 to 20 *μ*m from the interface, and then evolves up to about 2.5 *μ*m (at 200 *μ*m away the interface).

The picture changes significantly when the spinel powder obtained by the wet-chemistry route is used whereas the perovskite is still mechanosynthesized. In this case, the perovskite grain size increases continuously with distance and average sizes in the micron range are achieved at only 5 *μ*m from the interface (curve with closed dots in figure 5). Notably, both trilayers have basically the same average sizes at the interface and far away thereof, but its evolution with distance from the interface greatly differs. This is the result of the different sinterability of the phases involved, i.e., the delay in achieving high densification before the thermally activated processes for grain growth are triggered. A deeper insight into the underlying mechanisms can be obtained by studying the *in situ* shrinkage curves recorded during the SPS experiments, and the role of the different parameters on the microstructural features. A direct influence on the magnetoelectric response of multilayers obtained from these materials can be anticipated, attending to the functionality expected for these interfaces.

**Figure 6.**
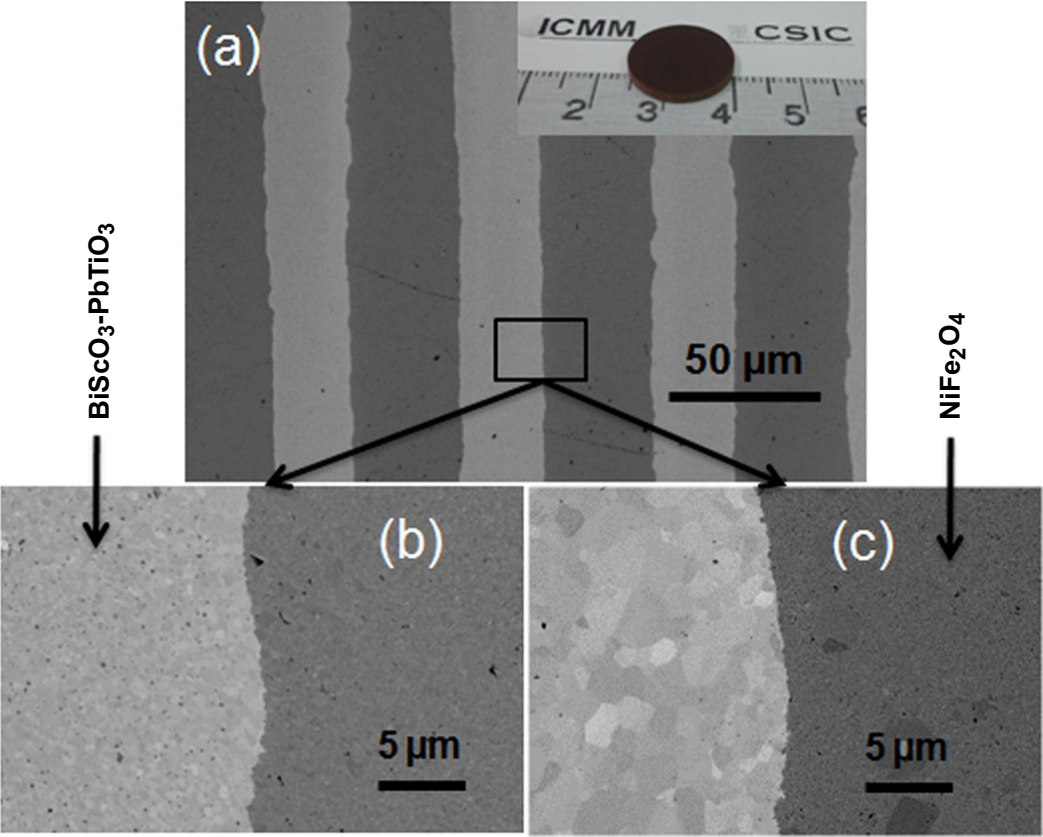
(a) Representative SEM micrograph of the cross-section of a multilayer structure made of alternating perovskite (light stripes) and spinel (dark stripes) phases by SPS at 900 °C. Images of the interfaces of multilayers prepared with ferrites obtained by (b) mechanochemical activation and (c) wet-chemistry.

Figure 6(a) shows a representative SEM micrograph of the cross-section of a multilayer structure made of alternating perovskite (light stripes) and spinel (dark stripes) phases by SPS at 900 °C. The approach is demonstrated suitable to obtain highly-dense microstructures with high-quality interfaces, avoiding thermal expansion mismatch between the two ceramics that usually results in cracks and delamination. The peculiar evolution of the perovskite grain size when the spinel is obtained by mechanosynthesis in trilayers does not occur similarly in multilayers, due to the small thickness of the layers. Note in figure 5 that similar grain sizes are obtained up to 30 *μ*m from the interface. In the multilayers, submicron grains are obtained across the whole perovskite layers. The different interfaces, basically in the perovskite grain size, of the multilayers prepared with the ferrites obtained by mechanosynthesis and wet-chemistry are shown in figures 6(b) and (c), respectively. Fairly dense microstructures were obtained in both cases at any distance from the interface. Improved properties would be anticipated for the latter, attending to the larger grain size near the interfaces.

**Figure 7.**
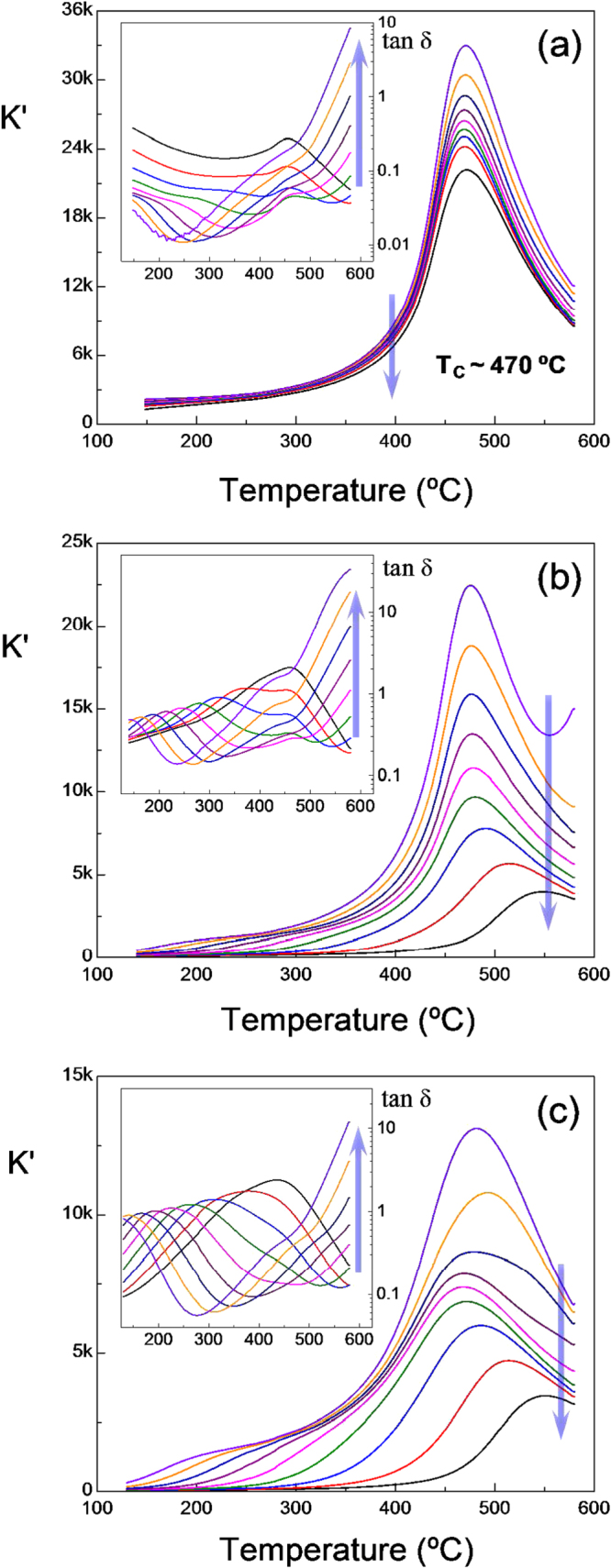
Relative permittivity *K*′ and dielectric losses (tan *δ*) as a function of temperature at several frequencies (0.1, 0.5, 1, 5, 10, 50, 100, 500, 1000 kHz; arrows indicate increasing frequency) for (a) the trilayer prepared at 900 °C with the ferrite obtained by wet-chemistry; and (b), (c) multilayers prepared at 900 °C with ferrites obtained by (b) wet-chemistry and (c) mechanochemical activation.

Figure 7(a) shows the relative dielectric permittivity *K*′ and losses (tan *δ*) as a function of temperature at several frequencies for the trilayer prepared at 900 °C with the ferrite obtained by wet-chemistry. *K*′ has been computed with the thickness of the piezoelectric element for comparison with the multilayers. Both *K*′ and tan *δ* curves hold a strong resemblance with the typical ones of BSPT ceramics [36], and indicate a very small influence of the spinel in the dielectric response of the trilayer. The anomaly associated with the ferroelectric transition is clearly observed at *T*_C_ ∼ 470 °C. The pronounced dispersion with frequency in *K*′ around the ferroelectric transition also resembles the previously reported data for BSPT ceramics, which was described as a grain boundary effect. Indeed, simulation of the electrical response for the grain bulk and boundary contributions shows the low-frequency data to be the closest one to the bulk response [37].

The dielectric response changes significantly for the multilayer composites. Figures 7(b) and (c) show the *K*′ and tan *δ* curves for multilayers prepared at 900 °C, using the ferrites obtained by wet-chemistry and mechanochemical activation, respectively. In both cases, the dielectric dispersion increases by a large extent, i.e., the maximum *K*′ decreases about 5 times on increasing frequency and shifts to higher temperatures for the highest frequencies. Note also the increase of the tan *δ* in more than an order of magnitude. This is not a grain boundary effect of the perovskite as described in trilayers, and neither a ferroelectric nor a relaxor-type phenomenon. This is most probably associated with a Maxwell–Wagner relaxation process that overlaps with the ferroelectric transition. This effect accounts for charge accumulation at the interfaces between the perovskite and the spinel components of the multilayers on the basis of the difference in the characteristic relaxation times in these two phases. Several relaxations would be expected by the presence of different thermally activated conductivity processes, i.e., electron hopping, oxygen vacancy displacements, etc.

Actually, the complex dielectric response of magnetoelectric composites with this 2–2 type connectivity has been described with Maxwell–Wagner relaxation model [38], in which the relaxation time was demonstrated that changed over a wide range of values by varying the volume fractions of the component phases. This effect must be also present in the trilayers, though in this case the perovskite phase dominates the dielectric response in the measuring frequency range. The change to a multilayer configuration with thinner piezoelectric elements and a larger number of interfaces modified the characteristic relaxation times and increases the Maxwell–Wagner extrinsic contribution to the dielectric response.

Nevertheless, the ferroelectric transition can be observed in the low-frequency data at the same *T*_C_ ∼ 470 °C for both multilayers. This is particularly clear for the multilayer prepared with the ferrite by wet-chemistry, as shown in figure 7(b), in which the lack of shift of the temperature of maximum *K*′ with frequency (in the low-frequency region) indicates it is the ferroelectric transition. Lower permittivity values were obtained for the multilayer prepared with the ferrite by mechanosynthesis, yet the transition is still visible as shown in figure 7(c). This sample showed perovskite grain sizes at the submicron range approaching the nanoscale. The most noticeable effect of decreasing the grain size of a high sensitivity piezoelectric ceramic into the submicron range, and further down to the nanoscale on its dielectric properties is a progressive broadening and depletion of the maximum in permittivity at the temperature of the ferroelectric transition [36].

**Figure 8.**
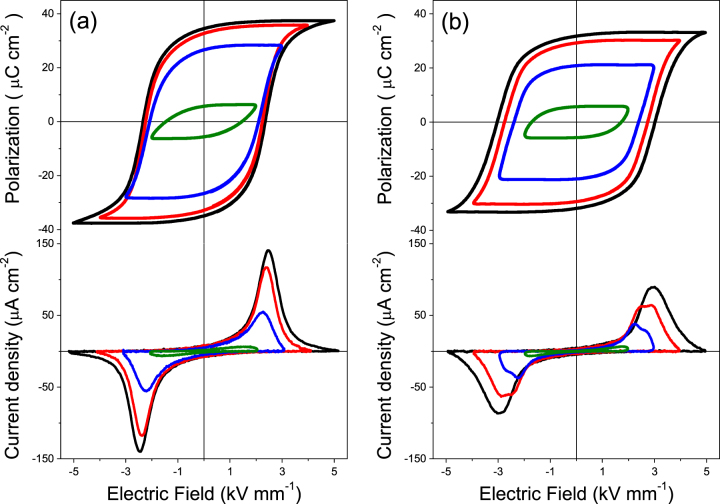
Ferroelectric hysteresis loops and current density curves with increasing applied electric fields for trilayers prepared at 900 °C with ferrites obtained by (a) wet-chemistry and (b) mechanochemical activation.

The ferroelectric hysteresis loops are typically good indicators of the ability for poling a ferroelectric material, and therefore useful to validate the achievement of an effective poling in the multilayer composites. It should be mentioned that the hysteresis loops usually reported for laminate composites with a direct bonding between the component phases resembles that of a lossy dielectric material with leakage currents [29]. Figure 8(a) shows the P–E curves along with their current density curves with increasing the applied fields up to 5 kV mm^−1^, for the trilayer prepared at 900 °C with the ferrite by wet-chemistry. This field amplitude is high enough to achieve a square loop and saturation of polarization, from which *P*_r_ of ∼35 *μ*C cm^−2^ resulted. The field evolution of the curves holds a strong resemblance with the typical ones of BSPT ceramics, for which a maximum *P*_r_ of ∼40 *μ*C cm^−2^ was achieved (see figure 3), indicating that the ferrite sandwiched between two perovskites behaves as a good conductive material, and highlighting the high quality of the interfaces achieved.

Figure 8(b) shows the P–E and current density curves for the analogous trilayer prepared with the ferrite by mechanochemical activation. In this case, square loops and saturation of polarization was also achieved, with a *P*_r_ of ∼32 *μ*C cm^−2^. However, the coercive field of the material increases from 2.3 kV mm^−1^ in the former case to 3.0 kV mm^−1^ in this last case, an increase that is attributed to the wide grain size gradient described in figure 5. This can be clearly observed in the current density curves, in which the evolution of the switching with increasing fields can be followed. Two peaks can be well differenciated on the current curves for applied fields of 3 and 4 kV mm^−1^, indicating that switching is not homogeneous. These peaks are attributed to the volume fractions of submicron-sized and micron-sized grains with different coercitivity in this material. The increase of the effective coercitivity as the grain size decreases was previously described. Finally, the curves evolve to achieve near saturation of polarization with a single but wider coercive field distribution for the highest applied field, dominated by the coercitivity of grains with the smaller sizes. The increase of the effective coercitivity may be a drawback to achieve an efficient poling of the composites.

**Figure 9.**
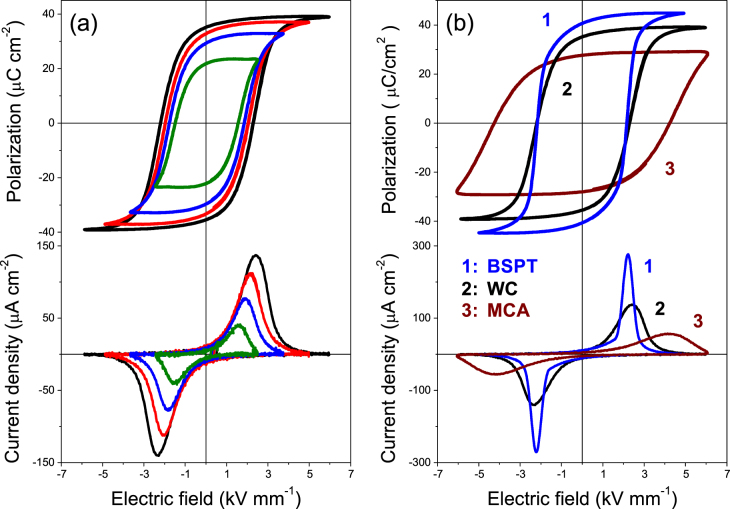
Ferroelectric hysteresis loops and current density curves for (a) a multilayer with the ferrite obtained by wet-chemistry; and (b) comparison between the coarse grained BSPT ceramic and multilayers with ferrites obtained by wet-chemistry (WC) and mechanochemical activation (MCA). All prepared by SPS at 900 °C.

Figure 9(a) shows the P–E and current density curves for the multilayer prepared at 900 °C with the ferrite prepared by wet-chemistry. Remarkably, the field evolution of the curves holds a strong resemblance with those of the analogous trilayer, saturation of polarization was practically achieved with the same *P*_r_ of ∼35 *μ*C cm^−2^ and *E*_c_ of 2.2 kV mm^−1^ as trilayers. The comparison of the hysteresis and their current curves between the multilayers prepared at 900 °C with ferrites by wet-chemistry and mechanochemical activation is shown in figure 9(b). The former (labelled **2** in the plot) shows values close to that of the coarse grained ceramic (labelled **1**), although the current density curve indicates a wider distribution of coercive field and thus of grain size, as expected. Note this material also presents submicron-sized grains at the interfaces, yet for a distance of only 5 *μ*m. On the other hand, the hysteresis loops for the latter (labelled **3**) resemble that reported for nanostructured BSPT ceramics [18]. The current curve indicates an extremely wide distribution of coercive fields associated with the small grain sizes, so higher fields are needed to achieve saturation. Nevertheless, a *P*_r_ of ∼28 *μ*C cm^−2^ was achieved, in good agreement with results in figure 3 for the similar grain sizes.

Poling was accomplished under the maximum field attained during loop measurements at a very low frequency (0.01 Hz), and after removing the field just before completing the loop. Berlincourt *d*_33_ coefficients were measured on the poled composites and included in table 1, which summarizes the macroscopic properties of the trilayers and multilayers processed at different synthesis and SPS conditions. In summary, composites prepared with the ferrites by wet-chemistry show higher *d*_33_ coefficients than those with the ferrite by mechanochemical activation, due to the better grain size distributions in the former. Besides, the higher the SPS temperature, the higher the *d*_33_ coefficient, as expected due to the increase in grain size. The *d*_33_ coefficients of the multilayers were three times lowers than those of trilayers under same synthesis and sintering conditions, despite them showing very similar hysteresis loops with full switching of the polarization. Besides, the best *d*_33_ for the composites were also lower than those for the BSPT ceramics with similar grain sizes. It should be taken into account that these coefficients should not be considered to be real values of the piezoelectric layers, but just considered as effective coefficients of the composites. It is difficult to know, for instance, which is the effect of the ac mechanical strain of the Berlincourt system on the ferrite layers, but they can be used for comparison between the different composites.

**Table 1.** Correlation between synthesis/processing conditions and macroscopic properties of trilayer (PSP) and multilayer (ML) ceramic composites. Berlincourt piezoelectric coefficient (*d*_33_) and maximum magnetoelectric voltage coefficient (*α*_31_) at the dc magnetic field (*H*_dc_).

Perovskite BiScO_3_–PbTiO_3_	Spinel NiFe_2_O_4_	SPS temp.	Comp.	*d*_33_ (pC N^−1^)	*α*_31_ (mV cm^−1^ Oe^−1^)	*H*_dc_ (Oe)
Mechanosynthesis	Mechanosynthesis	800 °C	PSP-trilayer	28 ± 3	5.0 ± 0.5	350 ± 50
		900 °C		230 ± 7	29 ± 3	255 ± 30
		1000 °C		240 ± 5	25 ± 1	200 ± 20
		900 °C	ML	65 ± 2	57 ± 1	245 ± 30
	Wet-chemistry	800 °C	PSP-trilayer	50 ± 3	12 ± 1	345 ± 45
		900 °C		300 ± 7	36 ± 3	235 ± 25
		1000 °C		315 ± 5	32 ± 2	190 ± 25
		900 °C	ML	105 ± 5	108 ± 1	205 ± 25

**Figure 10.**
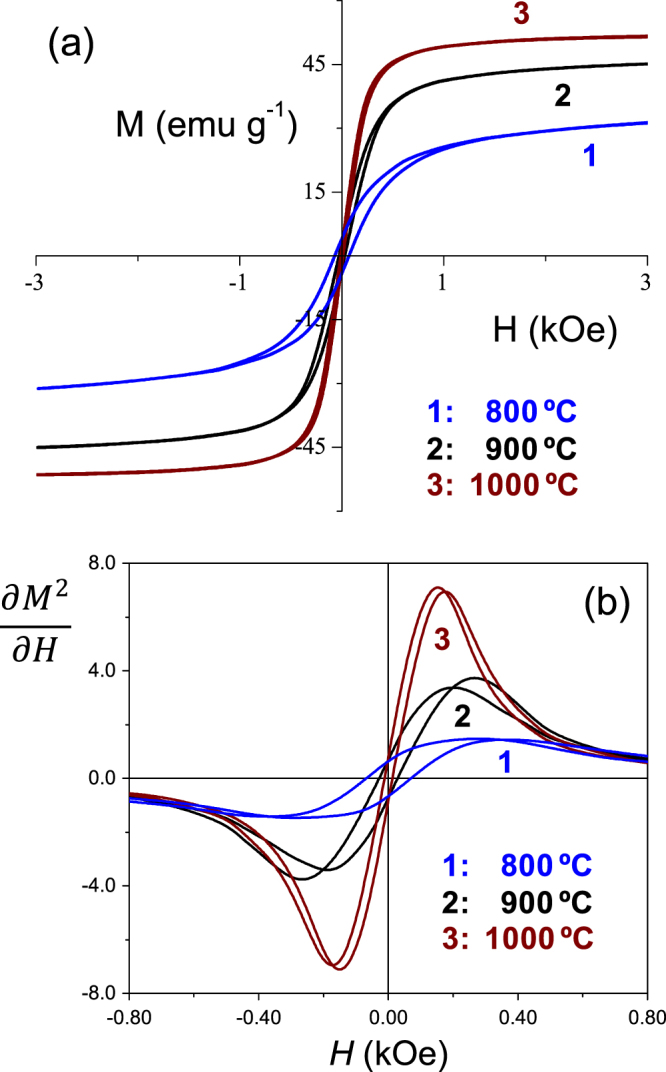
(a) Isothermal magnetization vs magnetic field loops and (b) derivative of square magnetization curves as a function of magnetic field, for trilayers prepared by SPS at different temperatures with the ferrite obtained by wet-chemistry.

The magnetization of the trilayers was also measured as a function of the magnetic field. Figure 10(a) shows the RT M–H hysteresis loops for the trilayer prepared at different SPS temperature with the ferrite by wet-chemistry. Similar data was obtained with the ferrite by mechanochemical activation. The saturation magnetization reaches the value of *M*_sat_ = 51 emu g^−1^ for the trilayer processed at 1000 °C, very close to that reported for bulk NiFe_2_O_4_ [39], and the remnant magnetization *M*_r_ and coercive magnetic field *H*_c_ were found to be 1.8 emu g^−1^ and 10 Oe, respectively. These are typical values for this ferrimagnetically ordered material, in which magnetic domain switched under very low applied magnetic field. With decreasing the SPS temperature two effects appear as a consequence of the reduction of the spinel grain size: (i) *M*_sat_ decreases and the material is increasingly far from reaching saturation of the magnetization, analogous to the results of the P–E curves in the perovskite, and (ii) *M*_r_ and *H*_c_ continuously increases, indicating that the spin canting contribution to the magnetization starts to dominate. *M*_r_ of 3 and 4.1 emu g^−1^ and *H*_c_ of 27 and 70 Oe were found for materials prepared at 900 and 800 °C, respectively. These results are in a good agreement with reports on NiFe_2_O_4_ nanocrystalline particles [39].

For magnetoelectric composites, the dependence of the magnetoelectric voltage coefficient (*α*_31_) on an applied dc magnetic field *H*_dc_ is tightly related with the effective piezomagnetic coefficient of the spinel phase obtained from the curve of in-plane magnetostriction *λ* as a function of *H*_dc_. The peak value in *α*_31_ is related to the position of maximum piezomagnetic coefficient [40]. However, some information can also be extracted from the magnetization hysteresis curves, for magnetostriction is correlated with the magnetization as *λ α* *M*^2^ [41]. A relation can be established between the low-field magnetization saturation behaviour and the position of the magnetoelectric *α*_31_ peak appearing at low magnetic dc field. Figure 10(b) shows the 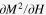 curves as a function of *H*_dc_ to illustrate the typical response that would be expected in the composites considering only the quality of the ferrite. Larger magnetoelectric response at lower *H*_dc_ would be expected with increasing the SPS temperature. Of course, the quality of the interfaces and the multilayers will influence the final response.

**Figure 11.**
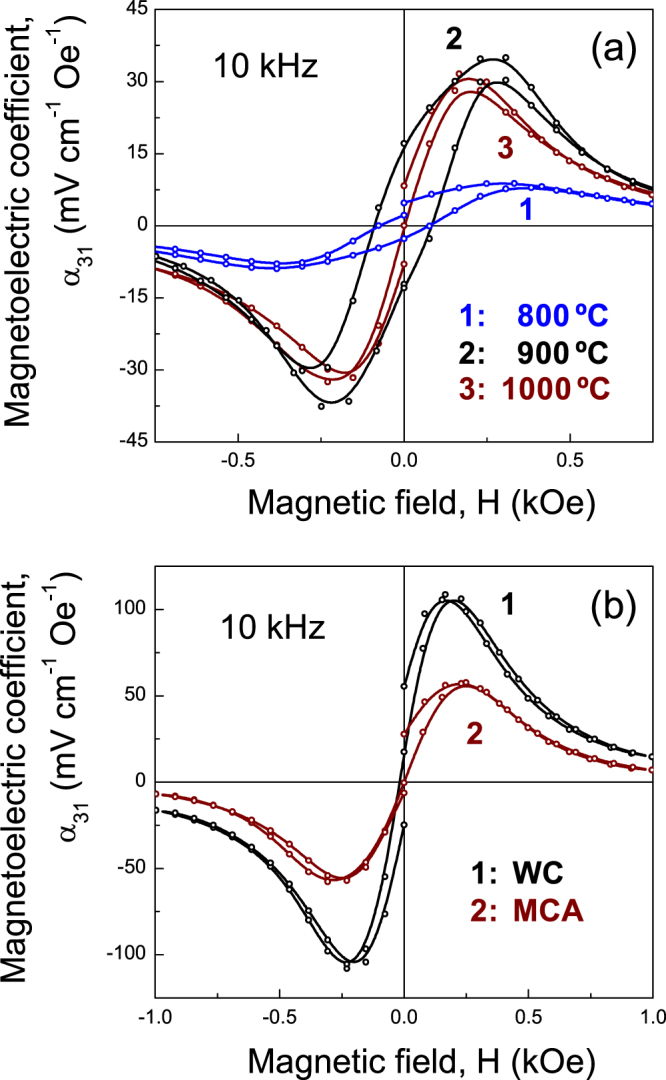
Magnetoelectric voltage coefficients (*α*_31_) as a function of dc magnetic field in the L–T mode for (a) trilayers prepared at different temperatures with the ferrite obtained by wet-chemistry, and (b) multilayers with ferrites obtained by wet-chemistry (WC) and mechanochemical activation (MCA).

Figure 11(a) shows the magnetoelectric voltage coefficients as a function of dc magnetic field in the longitudinal-transverse field mode (*H*_ac_ = 10 Oe at 10 kHz) of trilayers prepared at different temperatures with the ferrite by wet-chemistry. The curves were found to be closely related to the trend of those in figure 10(b), although the maximum *α*_31_ coefficient of about 35 mV cm^−1^ Oe^−1^ was obtained for the trilayer prepared at 900 °C. The peak voltage coefficient was obtained at relatively low dc fields (about 200–230 Oe), and increases with decreasing SPS temperature following the anticipated trend. Despite the fact that the inherent properties of the constituent phases for the trilayer at 1000 °C were better, the *α*_31_ coefficient was lower due to the poorer elastic coupling between two phases, emphasizing the role of the strain continuity across the interface. The *α*_31_ coefficient and the *H*_dc_ for the peak position in trilayers and multilayers are also included in table 1.

The *α*_31_ coefficients as a function of *H*_dc_ for the multilayers prepared at 900 °C with the ferrites by both wet-chemistry and mechanochemical activation, are shown in figure 11(b). In the first case *α*_31_ reaches up to 108 mV cm^−1^ Oe^−1^, which is almost double the value achieved for the latter. This improvement of the magnetoelectric performance can be attributed to the enhanced inherent properties of the perovskite, with a more homogeneous microstructure and tailored interfaces. The output voltage measured is lower than the direct magnetoelectric signal induced from the piezoelectric layers, because of the presence of the poorly insulating ferrite layers. Indeed, internal electrodes have been introduced between piezoelectric and magnetic layers, which can directly collect the output charges produced from the piezoelectric layers, and thus improve the magnetoelectric response [29, 42]. The results here presented demonstrate that direct bonding by SPS of the component phases without any internal electrode layer is suitable to obtain magnetoelectric composites.

## 4. Conclusions

This work demonstrates the suitability of the high-sensitivity MPB piezoelectric perovskite BiScO_3_–PbTiO_3_ in combination with the ferrimagnetic spinel NiFe_2_O_4_ for the preparation of laminate magnetoelectric composites with tailored interfaces. The use of the SPS technique and highly reactive nanopowders obtained by mechanochemical activation and wet-chemistry routes were the key to minimize chemical reactions at and interdiffusion across the interface. This low-temperature processing approach makes possible to obtain fully dense ceramic composites with high-quality direct bonding between the component phases. However, a significant grain size reduction also results, and thus, grain size effects on properties across the submicron range and approaching the nanoscale become an issue for functionality. Therefore, a compromise should be found between the benefit of a fine microstructure to decrease thermal expansion mismatch between the different components and the advantage of micrometer grain sizes to achieve efficient poling and good properties, which both determine the magnetoelectric voltage response. The most noticeable effect of decreasing the grain size of high sensitivity piezoelectrics into the submicron range is the increase of the effective coercitivity, a drawback to achieve an efficient poling of the composites. Once this compromise has been reached, a magnetoelectric coefficient *α*_31_ of 108 mV cm^−1^ Oe^−1^ has been obtained in multilayer composites, comparable to best values reported. The piezoelectric perovskite BiScO_3_–PbTiO_3_ is demonstrated to be especially suitable for cofired magnetoelectric composites owing to its better down-scaling behavior than Pb(Zr,Ti)O_3_.
